# Effect of Incorporating L-Shaped Folded Metal Aggregates on the Performance of Asphalt Mixtures

**DOI:** 10.3390/ma18133039

**Published:** 2025-06-26

**Authors:** Qingguo Yang, Kelin Chen, Longfei Guan, Ya Li, Yunhao Li, Yu Zhou, Wujing Yin

**Affiliations:** School of Civil Engineering, Chongqing Jiaotong University, Chongqiong 400074, China; 990020020405@cqjtu.edu.cn (Q.Y.); glf25810@163.com (L.G.); h13782706283@163.com (Y.L.); 622230970092@mails.cqjtu.edu.cn (Y.Z.); 622230951053@mails.cqjtu.edu.cn (W.Y.)

**Keywords:** L-shaped folded metal aggregates (LFMAs), asphalt mixtures, pavement performance, surface free energy

## Abstract

With the increase in heavy-load traffic and the growing frequency of extreme weather events, traditional rock aggregates, due to poor morphological stability, are unable to meet the performance requirements of high-grade asphalt pavements in complex environments. Most existing research on metal reinforcement focuses on fiber forms. This study innovatively introduces L-shaped multi-faceted metal aggregates (LFMAs). Through surface energy analysis and tests such as the Marshall test, rutting test, water immersion Marshall test, and freeze–thaw splitting test, the effects of the dosage and particle size of LFMAs on the performance of asphalt mixtures are explored. The results show that LFMAs can form an effective bond with SBS modified asphalt, improving the high-temperature stability and low-temperature crack resistance of asphalt mixtures. Under both water immersion and freeze–thaw conditions, the resistance of asphalt mixtures to water damage decreases with the increase in the dosage of metal aggregates. This research expands the application of three-dimensional metal aggregates, breaks through the limitations of fiber-based materials, and provides a new direction for the development of high-performance asphalt mixtures.

## 1. Introduction

With the increasing proportion of heavy-load traffic and the frequent occurrence of extreme weather, the performance degradation of asphalt pavement under complex working conditions has become increasingly severe. For example, acid rain can lead to the dissolution of alkaline components in aggregates, cause damage to the asphalt film, increase the void ratio of the mixture, and result in a substantial decrease in low-temperature performance, water stability, and fatigue resistance [1]. Under high-temperature and heavy-load conditions, the tilting and breakage of surface aggregates under loading represent a primary cause of reduced skid resistance [2]. As the core material of asphalt pavement structure, asphalt mixture contains aggregates accounting for up to 95% of its composition. The configuration of the skeleton structure formed by these aggregates directly determines the performance characteristics and service life of asphalt mixtures during road utilization [3]. The formation of this skeleton structure is governed by the morphological characteristics and spatial distribution of aggregates, making the study of aggregate morphological evolution mechanisms crucial for overcoming performance limitations in asphalt mixtures.

In recent years, researchers have established evaluation systems based on the distribution characteristics of aggregates to reveal the relationship between aggregate properties and asphalt mixture performance. Zou et al. [4] found that the aggregate structure has a significantly greater impact on asphalt mixture performance than coarse aggregate mod-ulus and gradation composition. Gao et al. [5] developed a quantitative characterization system for aggregate contact features utilizing X-ray CT three-dimensional reconstruction technology, through which they correlated the spatial distribution of aggregates with the shear strength and crack resistance of asphalt mixtures. Wang et al. [6] verified through tensile tests and fatigue experiments that the short-term and long-term mechanical properties of asphalt mixtures are directly influenced by aggregate contact characteristics. Re-search has shown that the correlation coefficients between aggregate morphological parameters (angularity, shape, roughness) and mixture mechanical performance are significantly higher than traditional aggregate strength parameters [7]. Cheng et al. [8] discovered that the deformation resistance of asphalt mortar and mixtures increases with enhanced aggregate morphological characteristics, particularly with coarse aggregates where the skeleton effect effectively strengthens deformation resistance. However, studies have found that incorporating elongated or flaky rock aggregates during compaction tends to cause fragmentation, thereby reducing asphalt mixture performance, demonstrating that the brittleness of rock aggregates imposes limitations on optimizing traditional rock material shape parameters [9,10].

Based on the aforementioned limits of shape parameters for rock materials, researchers have begun focusing on the impact of angularity and roughness on the performance of asphalt mixtures [11]. Chen et al. [12] found that the uniformity of asphalt distribution is significantly influenced by aggregate sphericity, angularity, and surface texture. Busang et al. [13] and Kuang et al. [14] discovered that angular and rough aggregates enhance interparticle interlocking, thereby improving load distribution and deformation resistance, which subsequently affects asphalt mixture performance. This microscopic interlocking effect has been validated at macroscopic levels. For instance, Valdés et al. [11] observed that aggregate morphology and surface texture influence mixture stiffness and crack resistance, with these effects being source- and processing-dependent. Ge et al. [15] employed molecular simulations to demonstrate that moderate surface roughness enhances interfacial adhesion energy, pull-out resistance, and fracture performance. Sun et al. [16] established that pavement surface characteristics are affected by aggregate angularity and roughness. However, numerous studies [17,18] indicate that while morphological optimization of rock aggregates improves mixture performance, inherent material properties lead to progressive angularity and roughness degradation during service, prompting re-searchers to explore innovative reinforcement system designs.

Current technical approaches for enhancing asphalt mixture performance primarily focus on two directions: (1) Asphalt matrix modification through specific additives that alter asphalt properties at microscopic levels [19,20]. For example, polymers like SBS modify molecular structures to enhance high-temperature stability by reducing thermal flow [21,22,23]. Basalt fibers reinforce mixtures through their high strength and modulus, constraining deformation while improving toughness and fatigue resistance [24]. However, these methods remain constrained by weak aggregate–asphalt interfacial bonding [25]. (2) Aggregate geometric optimization through gradation design and surface treatment to improve skeleton density, though conventional mineral aggregates face modulus limitations in heavy-duty applications [3,26]. Studies show that introducing metallic reinforcement phases can create stiffness–flexibility composite systems. Steel fibers enhance crack resistance through bridging effects [27,28,29]. Al-Ridha et al. [30] reported 34.2% and 36% improvements in Marshall stability for hot-mix asphalt with 0.2% steel fiber content under short- and long-term aging, respectively. Messaoud et al. [31] observed increased tensile strength, energy dissipation, and ductility modulus with steel fiber content, achieving optimal mechanical performance at 1% fiber content. Serin et al. [32] demonstrated that aluminum chips and iron powder additions in hot-mix asphalt significantly improve fracture energy peaks. However, existing metallic reinforcement research predominantly focuses on fibrous forms, lacking systematic investigation into 3D metallic aggregate inter-locking effects. Notably, compared to plastic-deformable metallic aggregates, conventional rock aggregates exhibit progressive angular wear and fragmentation under cyclic loading, with morphological stability declining significantly during service, directly affecting skeletal interlock structure and long-term pavement performance [33,34,35]. Metallic aggregates offer unique advantages through their plastic deformation capacity, enabling optimized stress transfer pathways when designed with three-dimensional spatial characteristics while leveraging inherent high strength and toughness.

In summary, current research gaps exist regarding metallic aggregates’ incorporation in asphalt mixtures, particularly concerning their impacts on skeletal structure and inter-facial adhesion with asphalt. Studies confirm that skeletal configuration and aggregate-bitumen adhesion constitute critical determinants of mixture performance [36]. Contemporary research typically evaluates pavement performance through high-temperature stability, low-temperature crack resistance, and moisture susceptibility [37,38,39,40], with inter-facial bonding characterized using boiling water tests and surface free energy theory [41,42]. This study employs surface energy theory to analyze interfacial adhesion characteristics, combined with Marshall testing, rutting tests, low-temperature bending tests, and freeze–thaw splitting tests to quantitatively assess how L-shaped metallic aggregates with varying dosages and geometries influence high-temperature deformation resistance, low-temperature crack resistance, and moisture stability. Furthermore, it investigates the effects of L-shaped metallic aggregate dimensions on skeletal structure and mechanical properties in asphalt mixtures.

## 2. Materials and Methods

### 2.1. Raw Materials

#### 2.1.1. LFMAs

**Figure 1:**
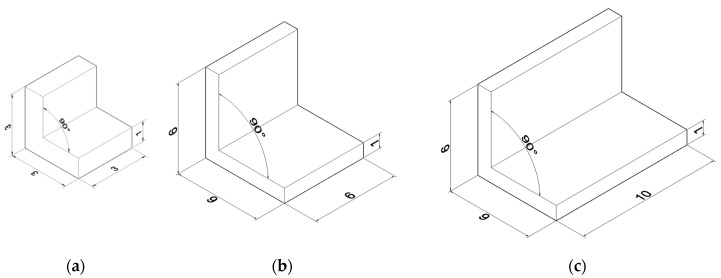
Geometric configurations of three different LFMAs (mm): (**a**) group L3; (**b**) group L6; (**c**) group L10.

The LFMAs used in the experiment were sourced from Zhonghao Metal Materials (Chongqing, China), made of 6061-T651 aluminum alloy. Figure 1 illustrates the geometric configurations of three different specifications of LFMAs employed in this study, while Table 1 summarizes their key physical and mechanical parameters.

Given that the metal surface was not roughened, the boiling water test specified in “Highway Engineering Asphalt and Asphalt Mixture Test Procedures” T 0616-1993 [43] was conducted to evaluate asphalt adhesion performance. The test results presented in Table 2 demonstrate that the adhesion grade of LFMAs in SBS-modified asphalt meets practical engineering requirements.

#### 2.1.2. Asphalt

Given the superior bonding performance of SBS-modified asphalt compared to unmodified asphalt, laboratory tests are conducted using SBS (I–D) modified asphalt. Basic properties are presented in Table 3, with all performance indices complying strictly with Test Methods for Asphalt and Asphalt Mixtures in Highway Engineering (JTG E20-2011) [43].

The selection of these two types of asphalt aims to investigate the impacts of combining different asphalt varieties with LFMAs on the performance of asphalt mixtures, providing data support for subsequent optimization of asphalt mixture properties.

#### 2.1.3. Aggregates and Fillers

In this study, limestone from Zhanfei Building Materials (Chongqing, China) was used as the aggregate, with limestone mineral powder serving as the mineral filler. The aggregate performance tests were conducted in accordance with the Test Methods of Aggregate for Highway Engineering (JTG E42-2005) [44] and the Test Methods of Bitumen and Bituminous Mixtures for Highway Engineering (JTG E20-2011). The fundamental properties of the coarse limestone aggregate are presented in Table 4, while the comprehensive technical properties of the fine aggregate are detailed in Table 5.

The technical performance indicators of the limestone powder filler all satisfy the relevant specification requirements. These high-quality aggregates and fillers establish a solid foundation for producing asphalt mixtures with stable performance.

### 2.2. Asphalt Mixture Design

**Figure 2:**
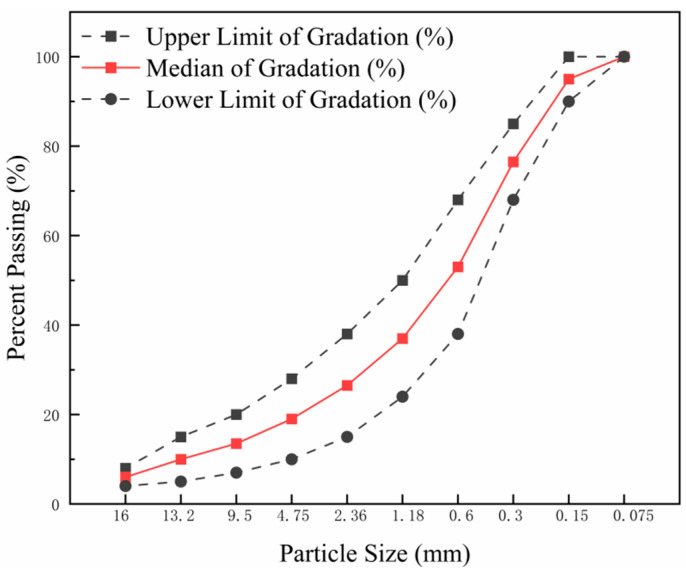
Gradation curve of aggregate, showing the relationship between particle size (mm) and percent passing, including upper limit, median, and lower limit of gradation.

This study employs AC-13 asphalt mixture prepared through a hot-mix process. The gradation design of the mixture adopts the median value of the recommended gradation range for AC-13 specified in the “Technical Specifications for Highway Asphalt Pavement Construction” (JTG F40-2004) [45]. The gradation curve is shown in Figure 2.

Based on a comprehensive literature review, detailed material characterization, and preliminary experimental results, the optimum asphalt content is determined to be 4.96%. Considering the unique spatial structure and performance characteristics of LFMAs, a three-gradient dosage system (1%, 3%, and 5% by mass) is designed. These aggregates are incorporated into asphalt mixtures via the external-addition method while preserving the original mineral-aggregate gradation.

### 2.3. Test Items and Experimental Methods

#### 2.3.1. Surface Energy Theory and Test Methods

(1) Surface Free Energy

Surface free energy is the additional energy possessed by surface molecules of a substance due to unbalanced intermolecular forces. Its dispersion component originates from instantaneous dipole interactions between molecules, while the polar component arises from polar interactions such as permanent dipoles or hydrogen bonds [41,42,43,45,46]. The two components satisfy Equation (1):

(1) $$\gamma =\gamma^{d}+\gamma^{p}$$

In the above-mentioned equation: γ—surface free energy (mJ/m^2^); $\gamma^{d}$—dispersion component of surface free energy (mJ/m^2^); and polar component of surface free energy (mJ/m^2^).

In asphalt mixtures, the adhesion between asphalt and aggregates arises from asphalt wetting the aggregate surface, a process accompanied by a reduction in the surface free energy of the asphalt–aggregate composite system. The interfacial free energy at this interface can be calculated using Fowkes’ theory, as shown in Equation (2):

(2) $$\gamma_{sL}=\gamma_{s}+\gamma_{L}-2\sqrt{\gamma_{s}^{d}\gamma_{L}^{d}}-2\sqrt{\gamma_{s}^{p}\gamma_{L}^{p}}$$

In the above-mentioned equation: $\gamma_{SL}$—surface free energy at the solid–liquid interface; $\gamma_{S}$—surface free energy of the solid; $\gamma_{L}$—surface free energy of the liquid; $\gamma_{s}^{d}$—dispersion component of the solid’s surface free energy; $\gamma_{L}^{d}$—dispersion component of the liquid’s surface free energy; $\gamma_{s}^{p}$—polar component of the solid’s surface free energy; and $\gamma_{L}^{p}$—polar component of the liquid’s surface free energy.

**Figure 3:**
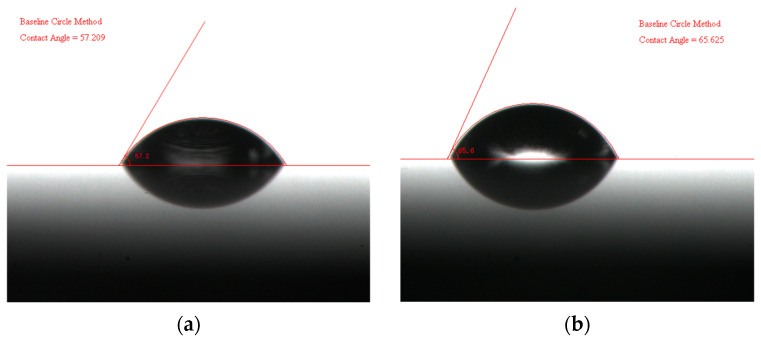
Measurement results of the water contact angle: (**a**) limestone; (**b**) aluminum alloy.

By integrating Young’s Equation (3), the contact angle *θ* of different liquids on the solid surface can be measured. As shown in the figures, Figure 3a illustrates the contact angle between limestone and water, and Figure 3b depicts the contact angle between aluminum alloy and water.

In this way, a linear Equation (4) can be established, which further enables the inverse calculation of the dispersion component (*γᵈ*) and polar component (*γᵖ*) of the aggregate for theoretical material selection guidance.

(3) $$\gamma_{sL}=\gamma_{s}+\gamma_{L}cos\theta$$

(4) $$\frac{\gamma_{L}(1+cos\theta )}{2}\frac{1}{\sqrt{\gamma_{L}^{d}}}=\sqrt{\gamma_{s}^{p}}\sqrt{\frac{\gamma_{L}^{p}}{\gamma_{L}^{d}}}+\sqrt{\gamma_{s}^{d}}$$

In the above-mentioned equations: $y=\frac{\gamma_{L}(1+cos\theta )}{2}\frac{1}{\sqrt{\gamma_{L}^{d}}};x=\sqrt{\frac{\gamma_{L}^{p}}{\gamma_{L}^{d}}},k=\sqrt{\gamma_{s}^{p}};b=\sqrt{\gamma_{s}^{d}}$.

(2) Adhesion Model

Surface energy theory defines the work of adhesion (*W_a_*) as the criterion for evaluating solid–liquid adhesion [47]. Under anhydrous conditions, asphalt and aggregates bond to form an asphalt–aggregate system, releasing energy equal to the composite’s *W_a_*. A higher *W_a_* indicates more energy release, a more stable state, and better adhesion.

The work of adhesion is calculated as follows in Equation (5):

(5) $$W_{as}=\gamma_{a}+\gamma_{s}-\gamma_{as}$$

In the above-mentioned equation: $W_{as}$—work of adhesion between asphalt and aggregates (mJ/m^2^); $\gamma_{a}$—surface free energy of asphalt (mJ/m^2^); $\gamma_{s}$—surface free energy of aggregates (e.g., LFMAs, $mJ/m^{2}$); and $\gamma_{as}$—interfacial free energy of the asphalt–aggregate system (mJ/m^2^).

#### 2.3.2. Marshall Test

The Marshall stability test is a key method for evaluating the mechanical properties of asphalt mixtures, primarily measuring their deformation resistance and load-carrying capacity at high temperatures. Conducted in accordance with T 0709-2011, specimens were submerged in a 60 ± 1 °C water bath for 35 min before loading on a Marshall stability testing machine at a rate of 50 mm/min, to determine Marshall stability (MS) and flow value (FL). The Marshall modulus (T), an indirect indicator of permanent deformation resistance, is calculated using Equation (6), showing consistency with the testing machine results:

(6) $$T=\frac{MS}{FL}$$

In the above-mentioned equation: T—Marshall modulus (kN/mm); MS—specimen stability (kN); and FL—corresponding flow value (mm).

#### 2.3.3. Rutting Test

**Figure 4:**
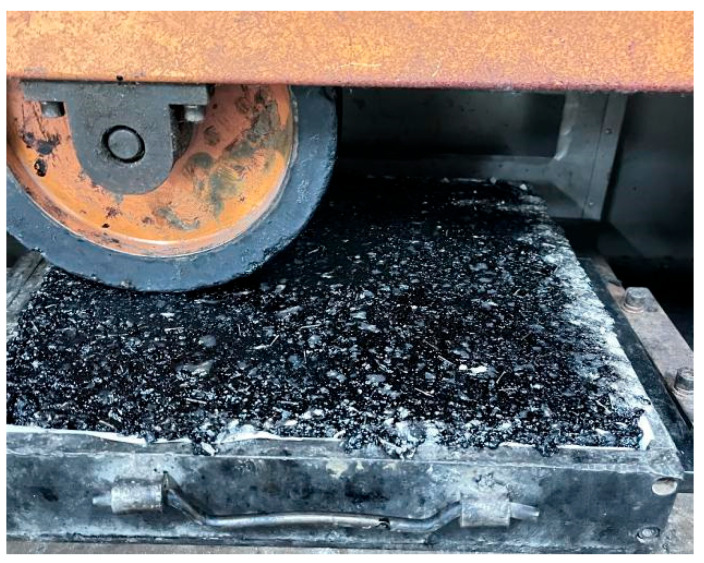
Hamburg wheel tracking device (HWTD).

The dynamic stability (DS) is used to evaluate the deformation resistance of asphalt mixtures under the repeated high-temperature loading of vehicles [40]. In this study, in accordance with the Chinese standard T 0719-2011, a Hamburg Wheel Tracking Device (HWTD), as shown in Figure 4, was employed to simulate the rutting conditions of actual road surfaces.

The test specimens, with dimensions of 300 mm × 300 mm × 50 mm, were tested under dry conditions at 60 degrees Celsius. A rubber wheel with a width of 50 mm and a thickness of 50 mm was used to apply a pressure of 0.7 megapascals at a frequency of 42 cycles per minute. Two parallel specimens were prepared for each group. The dynamic stability (DS) was calculated through Equation (7) based on the vertical deformation depths measured at 45 min and 60 min:

(7) $$DS=\frac{15N}{d_{60}-d_{45}}$$

In Equation (7): DS—dynamic stability of asphalt mixture (cycles/mm); N—wheel rolling speed (42 cycles/min); and $d_{60},\;d_{45}$—denote the vertical deformation depths of the asphalt mixture at 45 and 60 min of testing time, respectively (mm).

#### 2.3.4. Low-Temperature Crack Resistance and Test Methods

**Figure 5:**
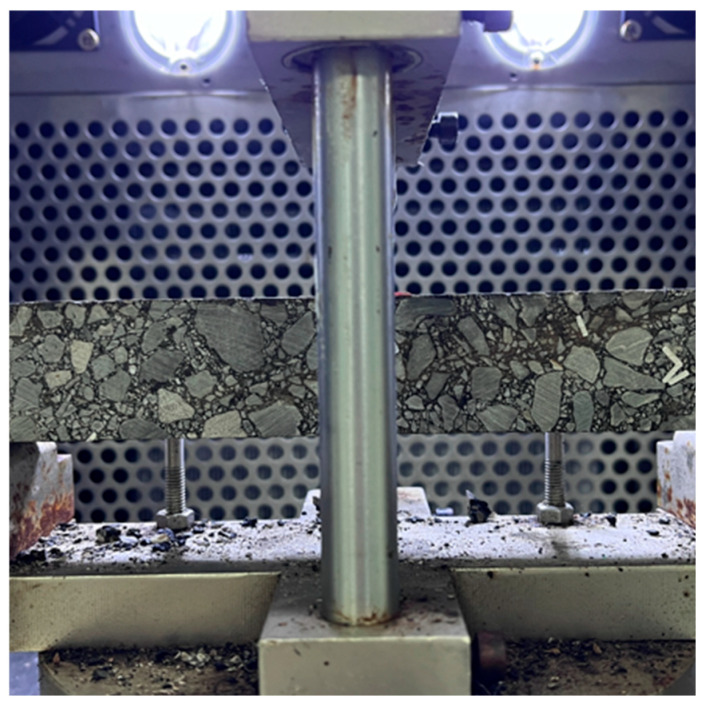
Specimens for the low-temperature small beam bending test.

The low-temperature crack resistance is an important index for evaluating the working performance of asphalt mixtures in low-temperature environments [37]. In this study, the low-temperature bending test was adopted to explore the low-temperature crack resistance of asphalt mixtures. According to the Chinese standard T 0715-2011, as shown in Figure 5, the size of the specimens for the bending test is 250 × 30 × 35 mm, and three parallel specimens are set for each group.

Firstly, place the specimens in an environmental chamber at −10 °C for 3 h. Then, use an MTS universal testing machine to test the middle part of the specimens at a constant loading rate of 50 mm/min. The evaluation indexes for the crack resistance of asphalt mixtures include the low-temperature flexural-tensile strength, flexural-tensile strain, and composite stiffness modulus, which can be calculated by the following Equations (8)–(10).

(8) $$R_{B}=\frac{P_{max}}{bh}$$

(9) $$\varepsilon_{B}=\frac{6h\delta }{L}$$

(10) $$s_{B}=\frac{R_{B}}{\varepsilon_{B}}$$

In the above-mentioned equations: $R_{B}$—low-temperature flexural-tensile strength (MPa); $P_{max}$—maximum failure load of specimen (N); b—specimen width (mm); h—specimen height (mm); $\varepsilon_{B}$—flexural-tensile strain (μm/m); δ—mid-span deflection of specimen (mm); L—specimen span (mm); and $s_{B}$—composite stiffness modulus (MPa).

#### 2.3.5. Water Stability and Test Methods

The Immersion Marshall Test and Freeze–Thaw Splitting Test analyze the difference in immersion stability and measure freeze–thaw splitting strength, respectively, to jointly establish a comprehensive evaluation system for the water stability of asphalt mixtures.

(1) Immersion Marshall Test

During actual road service, asphalt mixtures often suffer performance degradation due to water intrusion. Evaluating their moisture damage resistance includes two aspects: hot water damage resistance and freeze–thaw cycle damage resistance. In this test, Marshall specimens were divided into 4 groups with 3 parallel specimens each. Following Chinese standard T 0709-2011, specimens in three experimental groups were submerged in 60 °C water for 24, 48, and 72 h, respectively. Specimens (height 63.5 mm, diameter 101.6 mm) were tested using an HM-3000 main loader by Beijing Jingushenjian Measurement and Control Technology Research Institute (Beijing, China) at a loading rate of 50 mm/min. The evaluation index was retained Marshall stability, calculated via Equation (11):

(11) $${MS}_{0}=\frac{{MS}_{1}}{MS}\times 100\%$$

In the above-mentioned equation:${MS}_{0}$—retained Marshall stability (%); ${MS}_{1}$—Marshall stability after 48 h immersion (kN); and MS = Marshall stability after 30 min immersion (kN).

(2) Freeze–Thaw Splitting Test

In this test, Marshall specimens were divided into 2 groups with 4 parallel specimens each. Following Chinese standard T 0729-2011, specimens in the experimental group underwent freeze–thaw cycles: 16 h freezing at −18 °C in a constant-temperature refrigerator, followed by 24 h thawing in 60 °C distilled water. Control group specimens were stored at room temperature. Both groups were submerged in a 25 °C water tank for 2 h before testing their indirect tensile strength using an HM-3000 main loader at a loading rate of 50 mm/min. The resistance of asphalt mixtures to freeze–thaw cycle damage can be evaluated by calculating the freeze–thaw splitting tensile strength ratio (TSR), which can be determined using Equation (12):

(12) $$TSR=\frac{R_{T2}}{R_{T1}}\times 100\%$$

In the above-mentioned equation: TSR—freeze–thaw splitting tensile strength ratio (%); $R_{T2}$—splitting tensile strength of freeze–thawed specimens (MPa); and $R_{T1}$—splitting tensile strength of non-freeze–thawed specimens (MPa).

## 3. Results and Discussion

### 3.1. Interface Adhesion Characteristics Between Metal Aggregates and Asphalt

Contact angle and surface energy components are critical parameters characterizing material interfacial properties. This study selected 70# base asphalt (classified as Grade A road petroleum asphalt per JTG F40-2004, exhibiting 60–80 (0.1mm) penetration under T 0604-2011 standard conditions), along with SBS modified asphalt, limestone aggregate, and LFMA specimens, for contact angle measurements.

Results demonstrate that the coefficient of variation in the contact angle means for each material with three titration liquids—distilled water, glycerin, and formamide—is less than 5%, indicating good repeatability of the data in Table 6.

The high correlation coefficient of the linear fitting equation in Table 7 further verifies the reliability of the measurement method and the validity of the data.

According to the calculation results of surface energy and adhesion work in Table 8 and Table 9, the total surface energy of limestone is 47.67 $mJ/m^{2}$, and that of LFMA is 40.77 $mJ/m^{2}$, approximately 85.5% of limestone. The two show similar proportions of surface energy components, with dispersive and polar components being 26.04 $mJ/m^{2}$ and 14.73 $mJ/m^{2}$, respectively.

In addition, the work of adhesion between LFMAs and 70# asphalt, as well as SBS-modified asphalt, reaches 53.99 mJ/m^2^ and 58.80 mJ/m^2^, respectively. This indicates that their adhesion mechanism with asphalt is comparable to that of limestone. The current difference in adhesion work mainly stems from the higher total surface energy of limestone. In subsequent studies, surface modification can be employed to increase the proportion of polar components in LFMAs, further enhancing their interfacial bonding strength with asphalt.

### 3.2. Marshall Stability

According to Table 10, the reliability of the Marshall stability test results can be verified.

**Figure 6:**
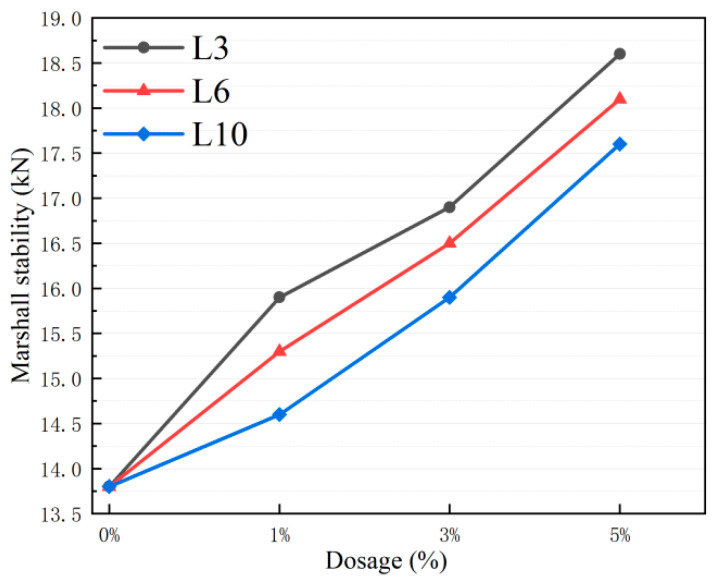
Marshall stability of asphalt mixtures with LFMAs.

As shown in Figure 6, the Marshall stability of the asphalt mixture with L-shaped corrugated metal aggregate is higher than that of the limestone asphalt mixture, and it shows a linear positive correlation trend as the dosage increases from 1% to 5%.

Among them, the L3-A5 group (with 5% of L3 aggregate added) performs the best, with a stability of 18.6 kN, which is 34.8% higher than that of the L0 group. The L6-A5 group (18.1 kN) and the L10-A5 group (17.6 kN) at the same dosage are 31.1% and 27.5% higher than the A0 group, respectively. It is worth noting that the stability of the L3 group under all dosages is better than that of the other aggregate size groups, and the performance improvement of the metal aggregate increases gradually as the aggregate size increases. According to Table 11, the flow values of the Marshall stability test are all within the engineering allowable range of 1.5–4.5 mm, and the coefficient of variation is less than 10%, indicating that the fluidity is up to standard and the data stability is excellent.

### 3.3. Dynamic Stability

**Figure 7:**
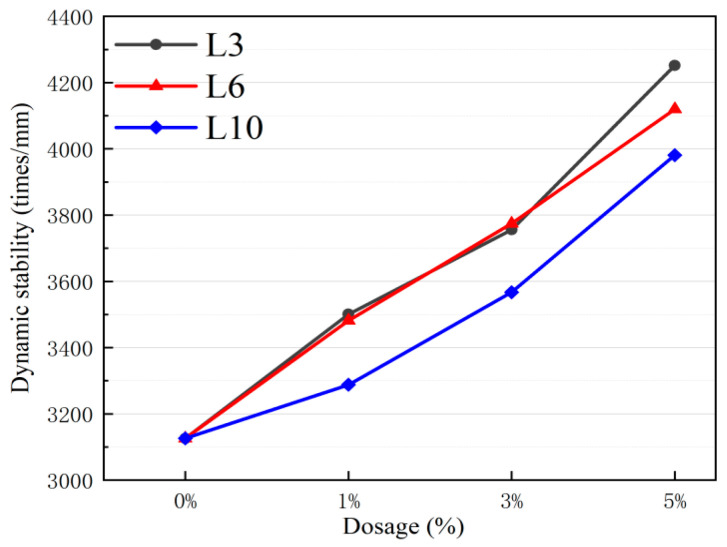
Dynamic stability of asphalt mixtures with LFMAs.

Dynamic stability characterizes the deformation resistance of asphalt mixtures under high-temperature conditions. Table 12 verified the reliability of the rutting test results. Figure 7 clearly indicates that the addition of LFMAs can enhance the high-temperature performance of asphalt mixtures, and there is a linear positive correlation as the dosage increases.

Among the different-sized L-shaped metal aggregate addition groups, the performance improvement of L3 metal aggregates is the most significant. Specifically, the L3-A5 group exhibits the highest enhancement in dynamic stability, reaching 4252 times/mm—a 36% increase compared to the control group L0 (3126 times/mm). In contrast, the L6-A5 group (4120 times/mm) and L10-A5 group (3981 times/mm) show improvements of 31.8% and 27.3% over the control group, respectively. Notably, at 1% and 3% dosages, the dynamic stability of the L3 group is comparable to that of the L5 group but remains higher than that of the L6 group.

### 3.4. Low-Temperature Cracking Resistance

Low-temperature small-beam bending test adopts the indicators of flexural tensile strength and flexural strain to, respectively, characterize the ability of asphalt mixture to resist bending tensile failure under low-temperature conditions, as well as the flexibility and crack resistance of the material.

Flexural Tensile Strength: the statistical analysis results in Table 13 effectively verified the reliability of the test data.

**Figure 8:**
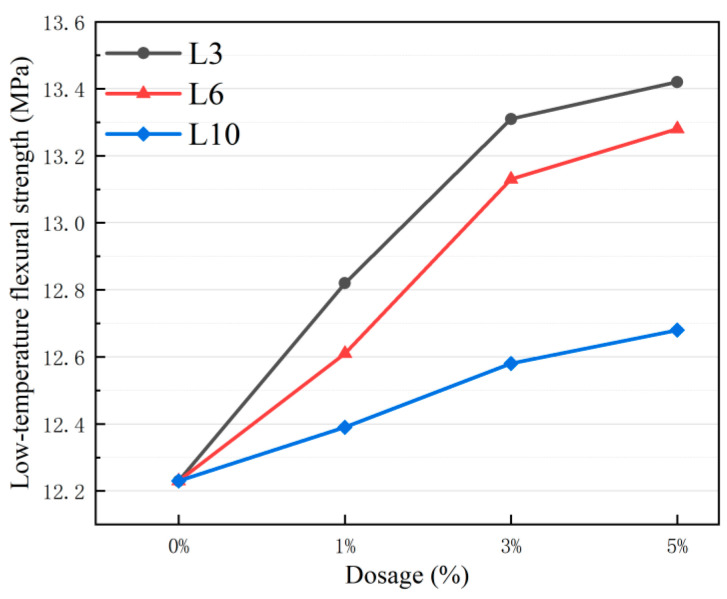
Low-temperature flexural-tensile strength of asphalt mixtures with LFMAs.

By combining the visualization presentation in Figure 8, it can be known that there is a linear positive correlation between the content of metal aggregates and the bending tensile strength of asphalt mixtures.

Moreover, in different L-shaped metal aggregate (LFMA) addition groups of varying sizes, the bending tensile strength of the L3 group at each content level exceeds that of the L6 and L10 groups. Specifically, the L3-A5 group demonstrates the most significant increase in bending tensile strength, reaching 13.42 MPa—a 10% improvement compared to the control group L0 (12.23 MPa). In contrast, the L6-A5 group (13.28 MPa) and L10-A5 group (12.68 MPa) exhibit 8.5% and 3.7% increases over the control group, respectively. Notably, the L3 group maintains better stability across all content levels than other aggregate size groups, and the performance enhancement of metal aggregates diminishes as aggregate size increases. Although the strength improvement of the metal aggregate asphalt mixture is relatively modest, the bending tensile strength indicators of all test groups surpass those of ordinary asphalt mixtures.

Maximum Flexural Tensile Strain: the statistical analysis results in Table 14 effectively verified the reliability of the test data.

**Figure 9:**
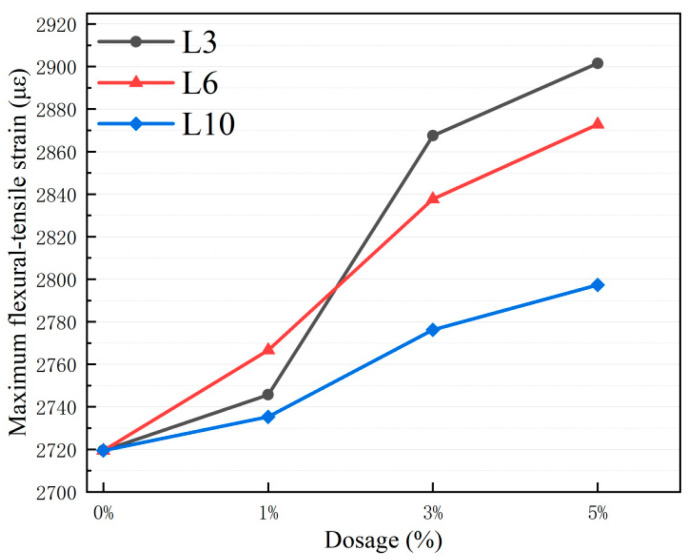
Low-temperature flexural strain of asphalt mixtures with LFMAs.

By combining the visualization presentation in Figure 9, it can be known that there is a linear positive correlation between the content of metal aggregates and the flexural tensile strength of asphalt mixtures.

Among the different L-shaped metal aggregate addition groups, the performance improvement effect of the L3 group is the most significant. When the content of the L3 group reaches 5%, the maximum flexural strain reaches 2901.5 με, which is an increase of 6.7% compared to the control group L0 with a maximum flexural strain of 2719.5 με. Meanwhile, the L6-A5 group (2872.8 με) and the L10-A5 group (2797.3 με) at the same content are 5.6% and 2.8% higher than the A0 group. Compared with other addition groups, the maximum flexural strain of the L3 group under all contents is higher than that of the L6 group and the L10 group. The maximum flexural strain of the L6 group gradually increases with the increase in the content, but the growth rate is smaller than that of the L3 group; the maximum flexural strain of the L10 group grows relatively more slowly and the overall value is lower than that of the L3 group. Although the increase in the toughness of the metal aggregate asphalt mixture is relatively limited, the toughness indicators of all test groups are greater than those of ordinary asphalt mixtures.

### 3.5. Water Stability

The Immersion Marshall Test and the Freeze–Thaw Splitting Test evaluate the water stability performance of asphalt mixtures under high-temperature and low-temperature conditions by analyzing stability differences during water immersion and measuring freeze–thaw splitting strength, respectively.

#### 3.5.1. Immersion Marshall Stability

**Figure 10:**
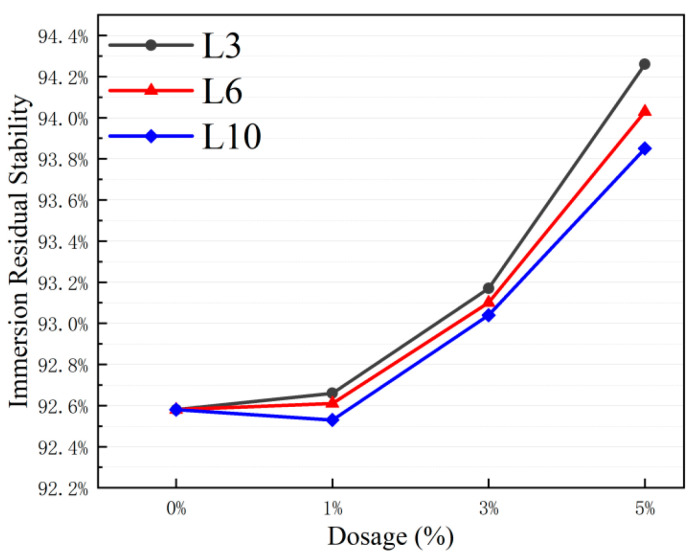
Immersion residual stability of asphalt mixtures with LFMAs.

The results of the water stability test of asphalt mixture are shown in Figure 10.

The residual stability of the mixture increases significantly with the increase in L-shaped metal aggregate content. Among them, the L3 group shows the best performance, with a residual stability of 94.26% at a 5% content, which is 1.68% higher than that of the control group A0. Across different particle size groups, the L3 group’s residual stability is 0.23% and 0.41% higher than those of the L6 and L10 groups, respectively. This improvement is attributed to the more uniform distribution of small-sized L3 aggregates within the mixture, which effectively fill voids and enhance inter-particle interlocking, thereby boosting the mixture’s water resistance.

Combining the statistical data in Table 15, the coefficient of variation in the Marshall stability before and after immersion water is less than 10%, and the standard deviation fluctuation range is between 0.43 and 0.91 kN.

This suggests that the test data in each group exhibits low dispersion and reliable results. A further analysis of stability changes before and after water immersion reveals that the average stability decrease after 48 h of water immersion is 6.8% compared to the 30 min measurement. The decrease in the test group with LFMAs is 3.2% lower than that in the control group A0. This verifies the inhibitory effect of metal aggregates on water damage in high-temperature environments from the perspectives of data stability and performance improvement magnitude.

#### 3.5.2. Freeze–Thaw Splitting Test

**Figure 11:**
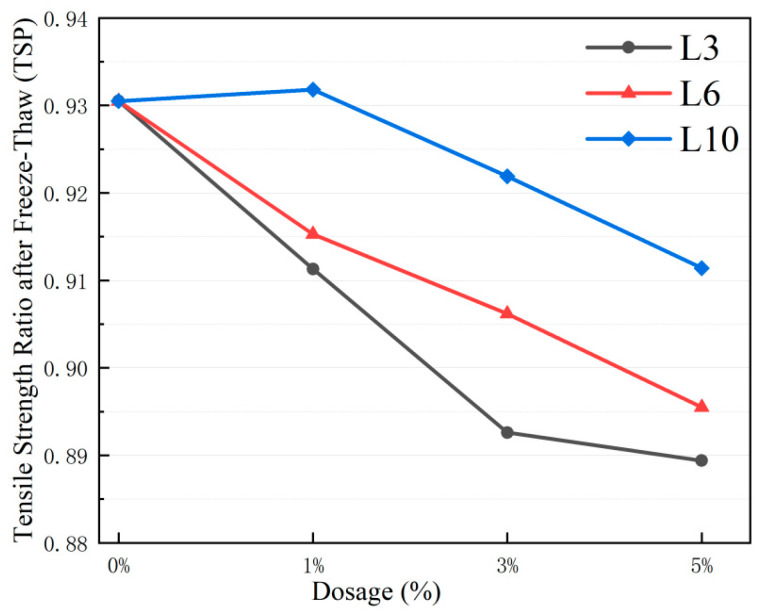
Freeze-thaw Tensile Strength Ratio of asphalt mixtures with LFMAs.

The results of the freeze–thaw splitting test of asphalt mixture are shown in Figure 11.

The freeze–thaw splitting strength ratio (TSP) decreases with the increase in L-shaped metal aggregate (LFMA) content. The control group L0 exhibits a TSP of 93.10%, which is higher than that of all mixed groups. Among different particle size groups, the L10 group demonstrates the best performance, with a TSP of 91.18% at a 5% content—2.30% higher than the L3 group and 1.52% higher than the L6 group. The L3 group shows the largest TSP reduction, with a 2.20% decrease at a 5% content compared to a 1% content. The interaction between particle size and content reveals that the L10 group has a TSP of 92.88% at a 1% content, showing no significant difference from the control group, while the L3 group has a TSP of 88.88% at a 5% content. This indicates that the freeze–thaw stability of metal aggregates is positively correlated with particle size and negatively correlated with content.

Based on the statistical data in Table 16 and Table 17, the coefficient of variation in the splitting strength before and after freeze–thaw is less than 5%, and the standard deviation fluctuation range is between 0.16 and 0.41 kN, indicating that the dispersion of the test data of each group is low and the results are reliable.

The mechanism analysis shows that the decrease in the low-temperature performance of the L-shaped metal aggregate–asphalt mixture is related to the interfacial adhesion performance. The small particle size metal aggregate may cause more damage to the asphalt–aggregate interface during freeze–thaw cycles due to its larger specific surface area, resulting in a TSP of 1.52% to 2.30% lower than that of the L10 group.

### 3.6. Comparative Analysis of Data

Through comprehensive analysis of the performance data of LFMAs with different particle sizes (L3, L6, L10) and dosages (1%, 3%, 5%) as shown in Table 18, the L3-A5 group with an L3 particle size and 5% dosage demonstrated superior comprehensive performance: in terms of high-temperature stability, its Marshall stability (MS) reached 18.6 kN and dynamic stability (DS) was 4252 times/mm, representing increases of 34.8% and 36% compared to the reference group A0, respectively—the highest values among all groups; for low-temperature crack resistance, the low-temperature flexural tensile strength was 13.42 MPa and the strain was 2901.5 με, with improvements of 10% and 6.7% relative to group A0, significantly enhancing crack resistance; and regarding water stability, its immersion residual stability of 94.24% was the best across all groups. Although the freeze–thaw splitting strength ratio of 88.95% was lower than some other groups, the L3-A5 group still exhibited overall advantages in high-temperature, low-temperature, and conventional water stability. Its balanced performance and significant improvements in key indicators outperformed other groups, making it an ideal choice for engineering scenarios with stringent requirements for high-temperature rut resistance.

**Figure 12:**
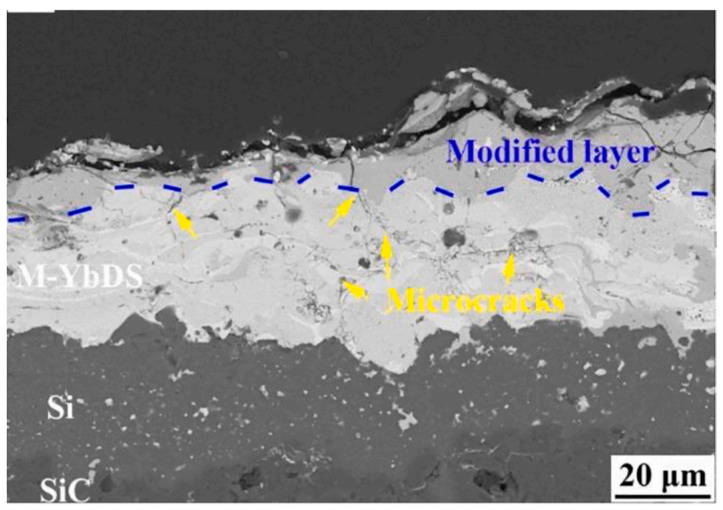
Cross-sectional structure of Al-modified M-YbDS coating [49].

Metal aggregates face significant corrosion risks in environments with water, road salt, and atmospheric moisture. For the aluminum metal used in this study, as shown in Figure 12, the dense aluminum oxide film formed on its surface after oxidation can effectively block Cl^−^ penetration [48,49]. Under salt spray corrosion, it exhibits only slight corrosion, with the corrosion potential shifting in a positive direction, significantly improving corrosion resistance. Moreover, after corrosion, the surface remains dominated by Al and O elements, maintaining structural stability. In contrast, untreated aluminum alloys suffer more severe corrosion damage due to the lack of a protective barrier.

Compared with traditional limestone aggregates, metal aggregates show unique ad-vantages in salt-corroded environments: limestone is affected by salt freeze–thaw cycles and is prone to physical dissolution and micro-crack propagation, leading to a decrease in immersion Marshall stability and a reduction in freeze–thaw splitting strength ratio [50]. Steel slag aggregates, due to containing active elements such as Fe and Ca, are prone to electro-chemical corrosion and alkali-silica reactions, resulting in a significant increase in porosity and structural degradation of the interfacial transition zone [51,52]. LFMAs relying on the physical barrier and chemical inertness of the oxide film exhibit better interfacial adhesion and freeze–thaw corrosion resistance than steel slag and limestone aggregates in salt environments. Combined with surface modification technologies such as sandblasting roughening and silane coupling agent treatment, their salt corrosion resistance can be further enhanced.

Through the comparative analysis of pavement performance with steel slag-incorporated asphalt mixtures in Table 19, the LFMA asphalt mixtures exhibit significant performance advantages [53]. Taking L3-A5 as an example, its Marshall stability reaches 18.6 kN and dynamic stability is 4252 times/mm, representing a 34.8% and 36% improvement over the reference group A0, respectively, highlighting outstanding high-temperature rut resistance. The low-temperature flexural tensile strength is 13.42 MPa with a strain of 2901.5 με, showing a 9.8% and 6.7% improvement, indicating better low-temperature toughness. The immersion residual stability is 94.24%, demonstrating good water stability. Although slightly lower than the reference group under freeze–thaw conditions, it outperforms steel slag-incorporated asphalt mixtures in high-temperature stability, structural bearing capacity, and low-temperature crack resistance.

## 4. Conclusions

This study addresses the demand for high-performance asphalt pavement materials by introducing LFMAs, and analyzes the pavement performance of asphalt mixtures compounded with granite aggregates and LFMAs of different sizes. The main findings are as follows:

1. LFMAs exhibit moderate surface energy, with their non-polar molecular structure generating polar forces due to the surface oxide layer. The adhesion work between LFMAs and SBS-modified asphalt reaches 58.80 mJ/m^2^. Although lower than that of limestone (62.60 mJ/m^2^), it still forms an effective interfacial bond.
2. The addition of LFMAs can increase the Marshall stability and dynamic stability of asphalt mixtures by up to 34.8% and 36%, respectively, and the flexural strength and strain at low temperatures by up to 10% and 6.7%, respectively. Its high-temperature and low-temperature performance show a linear growth with the increase in LFMA content. The enhancement effect is more significant under high-temperature conditions.
3. Under high-temperature conditions, the water stability of asphalt mixtures at high temperatures improves with the decrease in LFMA particle size and the increase in content. The addition of LFMAs in the L3-A5 group increases water stability by up to 1.68%.
4. Under low-temperature conditions, the water stability of asphalt mixtures in low-temperature environments decreases with the decrease in LFMA particle size and the increase in content. The addition of LFMAs in the L3-A5 group causes the maximum decrease of 4.1% in the TSP.
5. Limitations and future research

Although this study investigates the effects of incorporating LFMAs into asphalt mixtures on their pavement performance, certain limitations exist, necessitating further re-search:

1. In acid rain and coastal environments, the influence of the chemical reaction mechanism of the oxide film on the surface of L-shaped multi—folded metal aggregates (LFMAs) under environmental actions on the performance of asphalt mixtures.
2. The enhancement potential of LFMA’s surface modification and geometric parameters (e.g., particle size, fold structure) on mixture performance has not been fully exploited, warranting systematic research.
3. The impact of excessive shrinkage of LFMAs in low-temperature environments on the interfacial characteristics between asphalt and LFMA has not been considered and needs in-depth analysis.
4. During the construction and mixing process of LFMAs, local overheating is prone to occur, leading to a decline in asphalt performance.

## Figures and Tables

**Table 1:** Physical and mechanical parameters of LFMAs.

Measurement Parameter	Unit	Measured Value
Density (20 °C)	g/cm^3^	2.7
Melting Point	°C	582–652
Coefficient of Thermal Expansion (20–100 °C)	°C	23.6 × 10^−6^
Elastic Modulus	GPa	68.9
Poisson’s Ratio	-	0.33
Tensile Strength (25 °C)	MPa	310
Yield Strength (25 °C)	MPa	276
Hardness (500 kg force, 10 mm ball)	N/mm^2^	95

**Table 2:** Adhesion rating of LFMAs.

Asphalt	Aggregate Type	Asphalt Coverage Status	Asphalt Stripping Rate
SBS	L-shaped Folded Metal	Partial Asphalt Shedding	<5%
	Limestone	Asphalt Coverage Intact	0%

**Table 3:** Performance parameters of SBS-modified asphalt.

Material	Test Items	Unit	Result	Specification
SBS modified	Penetration (25 °C)	0.1 mm	51	40–60
Asphalt binder	Penetration Index	-	0.28	≮0
	Ductility (5 °C, 5 cm/min)	cm	28	≮20
	Softening Point	°C	81.3	≮60
	Viscosity	Pa·s	1.8	≯3
	Flash Point Temp	°C	293	≮230
	Elastic Recovery (25 °C)	%	86	≮75
RTPOT binder (163 °C, 75 min)	Penetration Ratio (25 °C)	%	70	≮65
	Ductility (5 °C)	cm	17	≮15
	Quality Loss	%	−0.52	≯±0.1

**Table 4:** Basic properties of coarse aggregates.

Index	Unit	Result	Standard	Method
Apparent Relative Density	13.2–16 mm	--	2.709	≥2.5
	9.5–13.2 mm	--	2.718	
	4.75–9.5 mm	--	2.714	
	2.36–4.75 mm	--	2.711	
Water Absorption	13.2–16 mm	%	0.56	≤3.0
	9.5–13.2 mm	%	0.58	
	4.75–9.5 mm	%	0.63	
	2.36–4.75 mm	%	0.79	
Los Angeles Abrasion Value	%	20.5	≤30	T 0317-2005
Flakiness and Elongation Index	%	8.7	≤20	T 0312-2005
Crushing Value	%	19.3	≤28	T 0316-2005
Adhesion with Asphalt	--	Grade 5	>Grade 4	T 0616-1993

**Table 5:** Technical property indices of fine aggregates.

Index	Unit	Result	Standard	Method
Apparent Relative Density	1.18–2.36 mm	-	2.660	≥2.5
	0.6–1.18 mm	-	2.679	
	0.3–0.6 mm	-	2.658	
	0.15–0.3 mm	-	2.616	
	0.075–0.15 mm	-	2.635	
Soundness (Part > 0.3 mm)	%	8.9	≤12	T 0317-2005
Content of Particles < 0.075 mm	%	0.9	≤1.0	T 0333-2000

**Table 6:** Contact angles of different specimens with three titration liquids.

Sample Type	Distilled Water (Mean °/CV%)	Glycerol (Mean °/CV%)
SBS	96.20/1.91	87.36/2.04
70#	102.65/0.84	91.92/2.07
Limestone	57.32/2.70	46.88/4.19
LFMAs	65.56/3.57	56.42/2.90

**Table 7:** Fitting equations of y-x and correlation coefficients for specimens.

Sample Type	Fitted Equation	Coefficient of Determination (R^2^)
70# Asphalt	y = 1.421x + 4.172	0.9551
SBS-Modified Asphalt	y = 1.757x + 4.459	0.9941
Limestone	y = 4.450x + 5.308	0.9984
LFMAs	y = 3.911x + 5.028	0.9426

**Table 8:** Surface energy calculation results of materials.

Specimen	Surface Energy (mJ/m^2^)	Dispersion Component (mJ/m^2^)	Polar Component (mJ/m^2^)
70# Asphalt	19.54	17.54	2.15
SBS Asphalt	22.86	19.84	3.02
Limestone	47.67	28.10	19.57
LFMAs	40.77	26.04	14.73

**Table 9:** Adhesion work between asphalt and aggregates.

Asphalt	Limestone (mJ/m^2^)	LFMAs (mJ/m^2^)
70#	57.37	53.99
SBS	62.60	58.80

**Table 10:** Statistical data of coefficient of variation and standard deviation for results of Marshall stability test.

Material Group	Mean Value of Marshal Stability (kN)	Standard Deviation (kN)	CV (%)
A0	13.8	0.2073	1.42%
L3-A1	15.9	0.0802	0.48%
L3-A3	16.9	0.0736	0.45%
L3-A5	18.6	0.1351	0.86%
L6-A1	15.3	0.2259	1.28%
L6-A3	16.5	4.0620	2.26%
L6-A5	18.1	0.6875	4.15%
L10-A1	14.6	0.1389	0.73%
L10-A3	15.9	0.527	2.80%
L10-A5	17.6	0.4375	2.35%

**Table 11:** Statistical data of coefficient of variation and standard deviation for results of Marshall flow value test.

Material Group	Mean Value of Marshal Flow Value (mm)	Standard Deviation (mm)	CV (%)
A0	3.55	0.0724	2.16%
L3-A1	3.42	0.045	1.15%
L3-A3	3.65	0.0719	2.01%
L3-A5	3.61	0.206	5.37%
L6-A1	3.75	0.0823	2.20%
L6-A3	3.66	0.09	2.26%
L6-A5	3.86	0.2486	7.10%
L10-A1	3.61	0.0861	2.28%
L10-A3	3.65	0.0512	1.22%
L10-A5	3.76	0.217	5.09%

**Table 12:** Statistical data of coefficient of variation and standard deviation for results of dynamic stability test.

Material Group	Mean Value of Dynamic Stability (times/mm)	Standard Deviation (times/mm)	CV (%)
A0	3126	82	2.63%
L3-A1	3501	79	2.26%
L3-A3	3756	136	3.63%
L3-A5	4252	128	3.02%
L6-A1	3482	111	3.18%
L6-A3	3775	108	2.87%
L6-A5	4120	111	2.69%
L10-A1	3288	106	3.21%
L10-A3	3567	104	2.91%
L10-A5	3981	106	2.67%

**Table 13:** Statistical data of coefficient of variation and standard deviation for results of low-temperature flexural tensile strength test.

Material Group	Mean Value of Flexural Tensile Strength (MPa)	Standard Deviation (MPa)	CV (%)
A0	12.23	0.15	1.21%
L3-A1	12.82	1.43	11.87%
L3-A3	13.31	0.52	3.97%
L3-A5	13.42	0.66	4.98%
L6-A1	12.61	0.8	6.38%
L6-A3	13.13	0.3	2.33%
L6-A5	13.28	1.42	12.13%
L10-A1	12.39	0.35	2.83%
L10-A3	12.58	0.98	8.44%
L10-A5	12.68	0.91	8.15%

**Table 14:** Statistical data of coefficient of variation and standard deviation for results of low-temperature flexural strain test.

Material Group	Mean Value of Flexural Strain (με)	Standard Deviation (με)	CV (%)
A0	2719.5	31.4	1.17%
L3-A1	2745.7	68.25	2.42%
L3-A3	2867.5	72.81	2.52%
L3-A5	2901.5	108.49	3.97%
L6-A1	2766.6	105.7	3.53%
L6-A3	2837.7	202.58	7.81%
L6-A5	2872.8	155.24	5.93%
L10-A1	2735.3	44.65	1.64%
L10-A3	2776.2	67.71	2.53%
L10-A5	2797.3	181.28	7.09%

**Table 15:** Statistical data of coefficient of variation and standard deviation for results of unimmersed Marshall stability test.

Material Group	Mean Value of Unimmersed Marshall Stability (kN)	Standard Deviation (kN)	CV (%)
A0	13.80	0.51	3.67%
L3-A1	15.10	0.66	4.37%
L3-A3	16.60	0.73	4.37%
L3-A5	17.70	0.73	4.10%
L6-A1	14.50	0.74	5.10%
L6-A3	16.23	0.78	4.77%
L6-A5	17.30	0.78	4.51%
L10-A1	14.20	0.91	6.41%
L10-A3	15.60	0.76	4.88%
L10-A5	16.80	0.74	4.41%

**Table 16:** Statistical data of coefficient of variation and standard deviation for results of splitting strength prior to Freeze–Thaw.

Material Group	Mean Value of Splitting Strength Prior to Freeze–Thaw (MPa)	Standard Deviation (MPa)	CV (%)
A0	1.278	0.35	2.84%
L3-A1	1.392	0.34	2.36%
L3-A3	1.468	0.37	2.45%
L3-A5	1.347	0.16	1.04%
L6-A1	1.435	0.37	2.66%
L6-A3	1.474	0.23	1.59%
L6-A5	1.396	0.30	2.00%
L10-A1	1.470	0.39	3.00%
L10-A3	1.525	0.24	1.69%
L10-A5	1.193	0.32	2.12%

**Table 17:** Statistical data of coefficient of variation and standard deviation for results of splitting strength after Freeze–Thaw.

Material Group	Mean Value of Splitting Strength After Freeze–Thaw (MPa)	Standard Deviation (MPa)	CV (%)
A0	1.191	0.35	3.05%
L3-A1	1.283	0.34	2.58%
L3-A3	1.338	0.37	2.75%
L3-A5	1.233	0.16	1.17%
L6-A1	1.300	0.39	3.06%
L6-A3	1.320	0.23	1.76%
L6-A5	1.272	0.30	2.24%
L10-A1	1.312	0.41	3.38%
L10-A3	1.356	0.22	1.69%
L10-A5	1.110	0.30	2.16%

**Table 18:** Statistical results of asphalt mixture tests with LFMAs.

Material Group	MS (kN)	DS (times/mm)	Mean Low-Temp Flexural Tensile Strength (MPa)	Mean Low-Temp Flexural Tensile Strain (με)	Immersion Residual Stability	TSR
A0	13.8	3126	12.23	2719.5	92.61%	93.06%
L3-A1	15.9	3501	12.82	2745.7	92.85%	91.13%
L3-A3	16.9	3756	13.31	2867.5	92.77%	89.26%
L3-A5	18.6	4252	13.42	2901.5	94.24%	88.95%
L6-A1	15.3	3482	12.61	2766.6	92.62%	91.53%
L6-A3	16.5	3775	13.13	2837.7	92.85%	90.63%
L6-A5	18.1	4120	13.28	2872.8	94.05%	89.55%
L10-A1	14.6	3288	12.39	2735.3	92.54%	93.14%
L10-A3	15.9	3567	12.58	2776.2	93.01%	92.16%
L10-A5	17.6	3981	12.68	2797.3	93.87%	91.17%

**Table 19:** Performance comparison between LFMAs and steel slag aggregate asphalt mixtures [53].

Material Group	MS (kN)	DS (times/mm)	Mean Low-Temp Flexural Tensile Strength (MPa)	Mean Low-Temp Flexural Tensile Strain (με)	Immersion Residual Stability	TSR
A0	13.8	3126	12.23	2719.5	92.61%	93.06%
L3-A1	15.9	3501	12.82	2745.7	92.85%	91.13%
L3-A3	16.9	3756	13.31	2867.5	92.77%	89.26%
L3-A5	18.6	4252	13.42	2901.5	94.24%	88.95%
L6-A1	15.3	3482	12.61	2766.6	92.62%	91.53%
L6-A3	16.5	3775	13.13	2837.7	92.85%	90.63%
L6-A5	18.1	4120	13.28	2872.8	94.05%	89.55%
L10-A1	14.6	3288	12.39	2735.3	92.54%	93.14%
L10-A3	15.9	3567	12.58	2776.2	93.01%	92.16%
L10-A5	17.6	3981	12.68	2797.3	93.87%	91.17%
AC-GSAM	12.7	2710	13.2	3120	84.90%	91.80%
AC-LAM	11.1	1480	12.8	2970	86.20%	82.30%
SMA-GSAM	9.1	3000	10.5	2820	87%	94%
SMA-LAM	8.4	2900	10.1	2640	88.50%	86.50%

## Data Availability

The original contributions presented in this study are included in this article. Further inquiries should be directed to the corresponding author.

## Abbreviations

The following abbreviations are used in this manuscript:

LFMA	L-shaped multi-faceted metal aggregate
SBS	Styrene–Butadiene–Styrene block copolymer
CV	Coefficient of variation
MS	Marshall stability
DS	Dynamic Stability
